# Co-development of a community pharmacy training regarding fentanyl and xylazine test strips

**DOI:** 10.1016/j.rcsop.2024.100557

**Published:** 2024-12-19

**Authors:** Grace Marley, Cheryl Viracola, Ainsley Bryce, Anthony Hudson, Elizabeth Locklear, Bayla Ostrach, Delesha Carpenter

**Affiliations:** a UNC Eshelman School of Pharmacy Division of Pharmaceutical Outcomes and Policy, USA; b Southeast Area Health Education Center, USA; c North Carolina Association of Pharmacists, USA; d Holler Harm Reduction, USA; e Roan Mountain Pharmacy, USA; f Rocky Point Pavilion Pharmacy, USA; g Fruit of Labor Action Research & Technical Assistance, LLC, USA; h UNC Eshelman School of Pharmacy, USA

**Keywords:** Community-pharmacy, Harm-reduction, Co-development

## Abstract

**Introduction:**

Fentanyl and xylazine test strips (FTS, XTS) are simple point-of-care tests that determine the presence of fentanyl or xylazine in a substance before use. Access to FTS and XTS is limited. For pharmacists who are willing to sell an FTS, there is little guidance about how to implement FTS sales and counseling as no training for community pharmacists regarding FTS and XTS exists. This article describes how a FTS and XTS training for community pharmacists was co-designed.

**Methods:**

A co-design strategy was utilized that involved an advisory panel of eight members: three practicing community pharmacists, two harm reduction experts, a website developer, the director of practice advancement for the state pharmacy association, and a patient-provider communication expert. A total of six meetings occurred to develop the training over seven months from July 2023 to February 2024. The advisory panel met once a month to discuss training goals, develop training information, and revise and structure the training to ensure the acceptability and appropriateness of the training for North Carolina community pharmacists.

**Results:**

The co-design strategy led to the development of a 6-module 30-min training. Module topics included information that stakeholders felt was most important to include: (1): What and Why of Test Strips, (2) Why pharmacies? (3) How to use/ “Best practices of testing” (4) Logistics (5) FAQs and (6) Resources. Panelists determined an online self-paced webinar would be most useful for pharmacists to reference when needed.

**Conclusion:**

The inclusion of stakeholders, including product end-users, leads to the creation of content that is salient and feasible for pharmacists to implement, which may increase their ability to integrate a new pharmacy service (FTS and XTS sales and counseling) into their pharmacy workflow.

**Patient or public contribution:**

This training was developed through a co-design strategy for community pharmacists with community pharmacist input. This training also utilized feedback from harm reduction experts who have trained people who use drugs on the best practices of testing their substances with FTS and XTS. The incorporation of their feedback was integral to the development of this training and will ensure that the training is feasible for the pharmacist to integrate into their workflow.

## 1 Introduction

According to provisional data, over 3300 people lost their lives in North Carolina (NC) due to fentanyl overdose in 2023.^1^ Fentanyl is a colorless, flavorless synthetic opioid that individuals may unknowingly consume in cases where it contaminates another substance without the person's knowledge. Similarly, xylazine is an emerging drug that may unknowingly contaminate other substances, contributing to the increasing number of drug overdose deaths as well as cause severe skin lesions that are painful and challenging to treat.^2^^,^^3^ Research has shown that 12–15 % of powder methamphetamine and powder cocaine samples sent to a drug checking service also contained fentanyl, and that the presence of xylazine reduced people who use drugs' ability to discern the presence of fentanyl.^4^ Fentanyl test strips (FTS) and xylazine test strips (XTS) are simple, point-of-care tests that can detect the presence of fentanyl or xylazine, respectively, in a non-prescribed substance before consumption. Previous studies on drug-checking test strips have shown their use to encourage safer drug use behaviors among people who use drugs (PWUD) such as using less of a substance, not using alone, and discarding a contaminated substance altogether.^5^, ^6^, ^7^ Equipping people to reduce the harms of xylazine use is of particular importance given the increasing rate of soft tissue infections resulting from unintentional xylazine exposure.^8^^,^^9^

Some states deem drug-checking equipment such as FTS and XTS drug paraphernalia and prohibit its distribution. A study of the legality of drug-checking equipment in the United States (US) found that it is legal to distribute drug-checking equipment (including FTS and XTS) in 30 states.^10^^,^^11^ In other states, laws were passed to allow for the distribution of FTS specifically, but do not currently allow for the distribution of XTS. Although FTS and XTS are accessible through harm reduction organizations such as syringe service programs in states where they are legal, this distribution is limited, especially in rural areas.^12^^,^^13^ Qualitative interviews of PWUD in Pennsylvania revealed a need for increased access to and awareness of drug-checking equipment, with respondents suggesting pharmacies as a potential location to receive FTS.^14^

Pharmacists currently engage in a variety of public health initiatives to counteract the opioid epidemic, including naloxone distribution, nonsterile syringe sales, and dispensing and counseling patients regarding medications for opioid use disorder.^15^^,^^16^ A survey of NC pharmacists found that most pharmacists were at least somewhat willing to sell FTS, distribute FTS instructions, counsel on FTS, and refer patients to harm reduction organizations when FTS were unavailable.^17^ This survey also found that pharmacists perceived that the sale of FTS would reduce overdose deaths; however, barriers to the implementation of FTS included a lack of awareness regarding where to order FTS and discomfort in initiating a conversation regarding FTS.^17^ A similar study of 376 Georgia community pharmacies found that only 54.7 % of pharmacies felt comfortable providing FTS education; however, 70.2 % of community pharmacists were willing to receive FTS information, indicating a need for training.^18^ A survey of rural southeastern US pharmacists found very limited knowledge of FTS and XTS; however, most pharmacists were willing to sell FTS and XTS.^31^

Currently, FTS and XTS training targeted to community pharmacy staff is nonexistent. Current trainings for pharmacy staff regarding reducing overdose harm emphasize naloxone dispensing and education, as well as discuss medications for opioid use disorder. Pharmacists have shown that training on naloxone dispensing leads to increased naloxone dispensing, and an improvement in pharmacists confidence in initiating a naloxone conversation.^41^^,^^42^ Similarly, a pilot study of a training to promote syringe sales by Arizona pharmacies found that staff reported an increased ease of dispensing syringes without a prescription after training.^43^ A content review of current pharmacist buprenorphine trainings found that although most trainings provided an overview of buprenorphine treatment, only 33 % of trainings identified addressed communicating with patients with buprenorphine prescriptions.^38^ Education regarding communicating with people who use drugs is important to overcome pharmacists fear of offending patients when discussing harm reduction strategies in the pharmacy.^39^^,^^40^ Person-centered communication training among pharmacists has shown to increase the odds of providing substance use disorder treatment.^37^

This article describes the process of working with expert advisers to develop a brief training for community pharmacy staff regarding FTS and XTS. The objective was to create a training module regarding the sale of FTS and XTS and the implementation of corresponding counseling in NC pharmacies. A co-design approach with expert engagement was chosen due to its ability to enrich the development, implementation, and evaluation of health interventions.^19^^,^^20^ The co-design strategy allows community and expert partners to provide input and collaborate to create a more relevant end product. In training development, this allows users to identify objectives and propose solutions to problems that they may have personally experienced. Participatory processes such as those used in this training are more effective in comprehensively identifying and addressing individual needs than non-participatory processes.^21^, ^22^, ^23^ Previous research has found that this approach is particularly helpful in creating innovative solutions to address community pharmacy-driven concerns and led to increased pharmacist willingness to engage with patients with opioid use disorder.^24^^,^^25^

## 2 Methods

A co-design approach was used to develop the FTS and XTS training titled “Test to Know”. The current project aimed to work with community pharmacy partners, harm reduction experts, an educational design specialist, health communication and pharmacy education researchers, and a website designer to implement drug-checking technology sales in community pharmacy settings. The development of this training followed the human-centered design process that provides a framework for moving quickly toward action through three phases: understanding, ideation, and implementation.^26^, ^27^, ^28^ This article details the iterative process of the co-creation of online training for community pharmacy staff in North Carolina. Distribution of FTS and XTS in NC community pharmacies is legal, and a previous study by Marley et al. (Jan 2023) indicated community pharmacist interest in receiving this type of training.^10^^,^^17^

An expert development panel comprising three community pharmacists, two harm reduction experts, one patient-provider communication expert one pharmacy practice advancement director with the NC Association of pharmacists, and a website designer helped develop and finalize material developed for the drug-checking pharmacy-based training. Panel members were purposively selected to ensure diverse perspectives on the training for NC community pharmacists. Specifically, three community pharmacists practicing in both western and eastern North Carolina were engaged in the project to ensure broad geographical applicability. Two harm reduction experts with experience dispensing and explaining the proper use of drug testing strips were engaged to ensure the accuracy of the training content, informed by use and distribution to people who use drugs. The director of pharmacy practice advancement with the state association of pharmacists was included to provide feedback regarding the ability to scale up the training statewide. A website designer ensured feasibility of website design elements proposed by other panel members. All panelists were invited via email to support the co-design process and had previous working relationships with the lead author (DC). The authors included in this study were panelists involved in the training design and reported no conflicts of interest. Panelists not already compensated as part of the grant funding the training development could opt to receive a $500 payment for their time.

Over seven months from July 2023 to February 2024, six development panel meetings were held in the late afternoon/evenings to accommodate busy pharmacy staff schedules. For participants who could not attend a specific meeting, meeting minutes were distributed afterward and feedback on module content was obtained via email. Topic materials and a meeting agenda were sent out the week before each meeting. The meetings occurred via Zoom to be most inclusive to panelists spread across the state, which also had the option to call in by phone for individuals who did not have reliable internet access. During all meetings, the facilitator took notes and recorded meeting minutes in real time utilizing a Google document. An outline of the meeting schedule and meeting content is provided in Table 1.

**Table 1:** Development panel engagement meetings and content outline.

Meeting	Number of attendees (including panelists and the moderator)	Topics covered
Meeting 1	8	● Reviewed formative study findings of pharmacist willingness to sell FTS and interest in training ● Elicited specific feedback on example web-based training that the research team previously created to determine the length of training and potential layout of training ● Decided on the length of the training (30 min), and training design (asynchronous website)
Meeting 2	8	Reviewed: ● Learning objectives for training ● How to divide the modules ● How to incorporate xylazine into the training module ● Proposed website content for module 1 and Module 2 including: ○ Pertinent information needed for pharmacists to know regarding what FTS and XTS are, and why they are important ○ Information regarding why pharmacists should get involved, including survey data indicating pharmacist willingness, and the impact pharmacists can have on access
Meeting 3	7	Reviewed: ● Proposed website content for module 3 and Module 4 including: ○ Content on how to test, and how to initiate the discussion of why patients should test ○ Content regarding how to interpret test strip results, with interactive feature ○ Content on the logistics of test strips including where to order, and where to place test strips in the pharmacy
Meeting 4	7	● Reviewed information to be included in Module 5: FAQs section, soliciting feedback on: ○ How to best incorporate legality information into the training ○ Best practices for communicating with patients about FTS/XTS ○ Additional information that patients should be given to accompany the test strips to ensure proper use
Meeting 5	8	● Reviewed existing test strip videos to determine best video content/format for training website ● Solicited feedback on example patient case videos and on interactive questions to include in training
Meeting 6	8	● Solicited feedback on the colors and interactive nature of the web-based training, including website navigation ● Reviewed final notes on training

*The content of the training was outlined and created by GM and reviewed by the study investigators and development panel. GM attended the meeting and took notes in real-time which were typed and reviewed with the study team for integration into the revised training content.

Before meeting with the development panel, the research team (GM, BO, DC) met to discuss the results from a formative study on North Carolina community pharmacists' willingness to sell FTS.^17^ Data from the formative study showed that pharmacy staff were willing to sell FTS, counsel on how to use FTS, distribute FTS, and refer patients to harm reduction organizations for FTS. ^17^ Pharmacists noted interest in training regarding FTS as well as barriers to selling FTS, which included not knowing where to order FTS and discomfort initiating a conversation about FTS. The survey data were summarized and presented over Zoom teleconferencing to the development panel during the first meeting. After receiving input regarding the panel's initial thoughts on what should be included in the training, the first author (GM) compiled and drafted the initial training content.

Meeting minutes were reviewed monthly by the study team, where suggestions for website improvements, usability, module language, and content were examined. Training materials were sent to panel members for feedback before meetings 4 and 6 so they could provide written feedback. During each meeting, all panelists were verbally asked for feedback on the training and areas for improvement.

Before the final panel meeting, the panel was sent an editable Google document with the co-designed training content, and additional feedback and approval were requested from each panelist. After feedback was obtained from the panelists, the facilitator incorporated feedback into an updated draft of the training, which then was reviewed by the study team during the final meeting. Verbal approval on the updated version was obtained from all study team members, at which the content was considered finalized. The resulting training module was then programmed by website development panelist.

## 3 Results

Six meetings occurred over the course of seven months, with an average of eight expert panelists. Each meeting lasted roughly one-hour and was facilitated over Zoom. Using the written notes from the panel meetings, changes to website content were integrated iteratively and reviewed by the panelists throughout the seven-month meeting period.

As this is the first web-based training for community pharmacy staff regarding test strip sales and counseling, the panel discussed the importance of keeping the training short (approximately 30 min), and prioritized information on what test strips are, how pharmacies can get involved, the logistics behind how to test, and how to implement this into practice. The panel decided training should be delivered asynchronously via a website to provide pharmacists with the ability to review the training at their own pace and at any time that is convenient to them. In addition to barriers identified by previous work, expert panelists believed the training should include content so that pharmacists fully understood how the test strips work, and how to efficiently communicate the use of drug checking equipment with patients.^17^

The panel prioritized content on logistics and how to incorporate test strips into the pharmacy workflow with minimal disruption to dispensing and other pharmacy services. The structure of the website was divided into six main sections: (1): What and Why of Test Strips, (2) Why pharmacies? (3) How to use/ “Best practices of testing” (4) Logistics (5) FAQs and (6) Resources. The panel wanted content that modeled realistic pharmacy interactions to increase the pharmacy staff member's comfort in initiating conversations about test strips.

### 3.1 Module 1: what and why of test strips

Pharmacy-based panelists believed the training should include information on the basics of drug-checking technology and increase pharmacy buy-in on selling test strips. Therefore, the first module opens with information regarding fentanyl and xylazine in the drug supply in NC, where it is found, and what happens with intake/overdose. They also wanted to include information about the accuracy and reliability of test strips, as well as how the test strips worked. Panel members mentioned that the average pharmacist may be unaware of xylazine and recommended including pictures of the adverse effects of xylazine to emphasize the impact that it could have as it is increasingly appearing in the local drug supply. The panel also recommended including information from previous studies of the experiences of PWUD and the positive effects of test strip use.

### 3.2 Module 2: why pharmacies?

Panelists stressed that they wanted to prioritize why pharmacists specifically should be engaged in test strip sales through Module 2.This module included information about where test strips are currently available in communities, and how access is limited, which could be overcome through increased pharmacy distribution of test strips. Additionally, information from the formative study showing that NC pharmacists are willing to engage in pharmacy sales was included in this module.^17^ Panelists mentioned that although it was important to prioritize increased access, the training would need to include information from a patient perspective. In order to incorporate this feedback, a video testimonial script was developed of a mother buying test strips for her son, because she is concerned that he does not know what is in the drug supply. This testimonial was based on and portrays a real-world experience a pharmacist-panelist reported of a conversation they had with a patient discussing FTS.

### 3.3 Module 3: best practices of testing

Video components in training have been found to increase self-efficacy in trainees and increase educational retention.^29^^,^^30^ Video examples of how to test drug samples utilizing FTS and XTS are included in Module 3 to provide visual supplementation to written information, and to increase educational retention. Video scripts were developed based on content from existing videos about test strips as well as input from harm reduction panel members. Panelists noted that they would like detailed information regarding the amount of water needed to use strips with different substances that are being tested, which was not covered in existing videos. Pharmacy panelists recommended including fliers or physical copies of instructions on the training website so they could print and share these with individuals who plan to utilize the test strips. They noted handouts would make it easier to incorporate education about test strips into their workflow. Lastly, in this section, panelists recommended creating a brief 2-min video that shows a pharmacist counseling a patient on FTS utilizing patient-centered, non-stigmatizing language. The script for this video was informed by community pharmacy staff and harm reduction panel members to ensure appropriateness and feasibility.

### 3.4 Module 4: logistics

Module 4 provides practical information for community pharmacists on where to order test strips and the differences between various brands of FTS and XTS available at the time the training was developed. Basic logistical information including where to obtain the test strips was included since a previous study determined that a primary concern among pharmacists otherwise potentially interested in dispensing them was not knowing how to order test strips.^17^ Panelists noted that as this would be a new practice in community pharmacies, some pharmacists may have difficulty determining where in the pharmacy the test strips should be placed. To overcome this, example information regarding where in the pharmacy FTS and XTS could be placed was added to the training.

### 3.5 Module 5: FAQs

The final module includes answers to questions that arose from the development panel. Information in this section is informed by pharmacist-panelists to answer questions that initially arose while actively considering how to incorporate FTS and XTS into their workflow. For example, it includes a video from the North Carolina Board of Pharmacy Executive Director affirming that NC pharmacists may distribute drug testing equipment without fear of legal recourse. In addition, this section has information on how to partner with harm reduction organizations, where to find up-to-date information about fentanyl and xylazine in the drug supply in North Carolina, and opportunities that pharmacists have to reduce overdose harm when unable to distribute FTS and XTS.

## 4 Discussion

Community member input has long been recognized as key to enhancing research validity and research applicability to patient and community-centered outcomes.^19^^,^^20^ The interdisciplinary study team engaged and co-developed the first training for community pharmacy staff regarding the sale and distribution of fentanyl and xylazine test strips. Through collaboration with community pharmacy staff members, harm reduction experts, and experts in pharmacy patient communication and community pharmacist training development, the real-world applicability of this training was ensured. The co-design strategy allowed for the creation of real-world video examples to model the implementation of test strip sales and counseling in a community pharmacy setting. Similar to other co-design training approaches, the study team found that by actively engaging community experts at each stage of the design process, training content was developed to be realistic and relatable to the needs of pharmacy staff.

Lack of access to FTS and XTS limits the impact of drug checking test strips.^33^ Pharmacists have expressed in various surveys that they are willing and interested to engage and improve access to drug test strips despite having limited knowledge about test strips.^17.18.32,33,35^ This training was developed to overcome pharmacist cited barriers to implementation, including lack of knowledge regarding drug checking strips, discomfort initiating a conversation about test strips, and concerns regarding logistics and pharmacy workflow.^17^^,^^34^ Although most panelists believed that charging a small fee for the drug checking test strips would allow for the integration of FTS and XTS distribution at the pharmacy to be sustainable, most distributors of FTS and XTS charge no cost to the FTS/XTS recipient.^32^ Additional research is needed to evaluate consumer willingness to pay for FTS and XTS, as well as PWUD's perceived willingness to obtain FTS and XTS from the community pharmacy.

For communication-related content, pharmacists expressed a need for training content on how to confidently initiate a conversation about FTS in an efficient manner so they could implement it in their busy workflows. A short video modeling a patient-centered non-stigmatized interaction between a pharmacist and test strip buyer was developed to improve pharmacist comfort initiating the conversation about test strips. Studies have found that the use of patient-centered communication increased patient satisfaction, treatment adherence, and improved medical outcomes.^35^ Other research has shown that brief trainings similar to this have increased pharmacist confidence discussing sensitive topics such as drug use, and an increase in confidence leads to an increase in pharmacist information provision.^36^^,^^37^ Future work should evaluate if pharmacists' confidence to initiate a drug checking conversation with consumers changes after completion of the training.

### 4.1 Strengths and limitations

The co-design approach provided a framework to gather development panel feedback, and receive iterative input on training content during each meeting. Expert panelists advised on the training content, wording, and delivery of each module. The way that panel members were engaged during and between meetings allowed for the development of content that was feasible to implement at the pharmacy.

The online format of meetings allowed for active participation of panel members at times that were convenient for them to meet. Despite the use of an online format, trust between members was built throughout the meetings and panel members were comfortable expressing dissenting opinions about content. The use of a Google document allowed for real-time revisions, and allowed timely integration of feedback into the training, without the challenges of everyone working asynchronously off different documents.

Providing deadlines for feedback and the monthly cadence of meetings allowed for the development of a comprehensive training in just seven months. One lesson the team has learned is to be flexible with pharmacist time, as oftentimes schedules can be challenging to predict in community pharmacy settings. Through this flexibility, if pharmacists were unable to meet at scheduled times, feedback was solicited after the meeting to inform the development of the training. Thus, everyone's feedback was incorporated even when they were unable to attend an online meeting.

Careful incorporation of feedback from the North Carolina Board of Pharmacists (NCBOP) was important to ensure that there was no misinterpretation of the law or legality associated with FTS and XTS sales in North Carolina. Panelists in the development of this training thought it was important to briefly mention that FTS and XTS sales were legal, and include a brief video from the executive director of the NCBOP detailing the legality of the test strips. Drug-checking equipment, such as test strips, is clearly legal in 30 states.^10^^,^^11^ In an 16 additional states, the distribution of FTS is legal; however, in these states, laws are specific to the distribution of FTS and do not include XTS.^11^ This proposed training guide provides valuable information informed by community experts that should be tailored for the practice area in each state to ensure legal safeguards are in place. Through the distribution of FTS and XTS in community pharmacies, pharmacists can engage patients who may otherwise feel stigmatized, and can help increase access to this harm reduction supply, especially for rural underserved populations.^17^ Further legislative work needs to be done to expand drug-checking equipment laws to reduce the harm associated with drug use, as well as allow for the expansion of this training beyond North Carolina community pharmacists.

The lack of pharmacy technician input in the development of this training cannot go unmentioned. Pharmacy technicians and cashiers are typically the first individuals who interact with patients at the pharmacy and would likely be the individuals physically selling the test strips. During the development of this training, the importance of ensuring technician and cashier support for the distribution of FTS and XTS was discussed. This support could be garnered through pharmacists directly discussing FTS and XTS with their staff. Future work is needed to evaluate the implementation of FTS and XTS at the pharmacy counter, with an emphasis on technician and cashier perspectives.

The dissemination of this training will occur in the coming year after the videos have been recorded, edited, and added to the study website. The videos are important to model how to use test strips, provide a “patient perspective”, as well as demonstrate how to counsel about test strips. Future work will be done to evaluate how pharmacist knowledge, confidence and perspectives surrounding FTS and XTS change after completion of the training. Three months after dissemination of the training, additional work will be done to evaluate the impact of this training on pharmacist practice, including evaluating whether the training led to an uptake of FTS and XTS sales in NC pharmacies.

## 5 Conclusion

A brief 30-min online training for community pharmacy staff about fentanyl and xylazine test strips was developed. The co-design methodology with community experts was vital for ensuring that the training was feasible and applicable to pharmacy staff. The implementation of fentanyl and xylazine test strips at community pharmacies has the potential to drastically increase access to this important harm-reduction tool, and potentially save lives. Future work will be done to develop the videos for this training and disseminate the training to practicing North Carolina community pharmacists.

## Funding

This project was supported by a 2023 Tar Heel Bus Tour Grant through Carolina Across 100.

## CRediT authorship contribution statement

**Grace Marley:** Writing – original draft, Visualization, Project administration, Methodology, Formal analysis, Data curation, Conceptualization. **Cheryl Viracola:** Writing – review & editing, Investigation. **Ainsley Bryce:** Writing – review & editing, Investigation. **Anthony Hudson:** Writing – review & editing, Methodology, Investigation. **Elizabeth Locklear:** Writing – review & editing, Methodology, Investigation. **Bayla Ostrach:** Writing – review & editing, Methodology, Investigation, Funding acquisition, Conceptualization. **Delesha Carpenter:** Writing – review & editing, Project administration, Methodology, Investigation, Funding acquisition, Data curation, Conceptualization.

## Declaration of competing interest

The convening of the advisory group was supported by a 2023 Tar Heel Bus Tour Grant through Carolina Across 100. The authors do not have conflicts to disclose.
